# LINE-1 Retrotransposons and Amyotrophic Lateral Sclerosis

**DOI:** 10.3390/ijms27146244

**Published:** 2026-07-14

**Authors:** Tinkara Korošec, Boris Rogelj, Vera Župunski

**Affiliations:** 1 Department of Biochemistry, Faculty of Chemistry and Chemical Technology, University of Ljubljana, Večna pot 113, 1000 Ljubljana, Slovenia; tk70242@student.uni-lj.si (T.K.); boris.rogelj@ijs.si (B.R.); 2 Department of Biotechnology, Jožef Stefan Institute, Jamova 39, 1000 Ljubljana, Slovenia

**Keywords:** amyotrophic lateral sclerosis, LINE-1, retrotransposons, TDP-43, cGAS–STING, neuroinflammation, epigenetic dysregulation

## Abstract

Amyotrophic lateral sclerosis (ALS) is a fatal neurodegenerative disease characterized by the progressive degeneration of upper and lower motor neurons. While monogenic causes account for a minority of cases, in most cases, ALS is sporadic and likely arises from multilayer interactions of genetic architecture, aging-associated loss of genome regulation, and inflammatory stress. Long interspersed nuclear element-1 (LINE-1) retrotransposons are endogenous mobile elements that are tightly controlled through various cellular mechanisms under normal conditions. When abnormally active, they are involved in gene inactivation, expression regulation, and genomic instability, leading to cellular processes such as innate immunity and cell death. Here, we present mechanistic links between LINE-1 and ALS. These include evidence that the burden of retrotransposition-competent LINE-1s (RC-L1s) is increased in ALS genomes, positioning RC-L1 load as a candidate contributor to missing heritability in sporadic disease. We also integrate emerging data showing that LINE-1 RNA can be intrinsically toxic independently of new insertions, as it promotes chromatin opening and transcriptional epigenetic noise, particularly when nuclear RNA surveillance pathways fail in TDP-43 pathology. Finally, we review how LINE-1-derived DNA/RNA intermediates can engage innate immune sensors, highlighting the cGAS–STING axis as a plausible route from LINE-1 de-repression to neuroinflammation. Together, these concepts support a model in which genetic RC-L1 load and age-/pathology-driven LINE-1 de-repression converge on nuclear dysfunction and inflammatory amplification, suggesting concrete molecular nodes for therapeutic intervention.

## 1. Introduction

In recent years, growing evidence has linked neurodegenerative disorders, including amyotrophic lateral sclerosis (ALS), to increased expression and mobilization of the human retrotransposon Long Interspersed Nuclear Element-1 (LINE-1). Therefore, in this review, we first provide an overview of ALS and the biology of the LINE-1 retrotransposon and then discuss the molecular mechanisms that restrict LINE-1 activity in the host, its physiological roles in the nervous system, and the evidence linking LINE-1 dysregulation to ALS. Finally, we evaluate the therapeutic potential of targeting LINE-1 activity and highlight key challenges and future directions for research in this emerging field.

ALS is a fatal neurodegenerative disease driven by progressive loss of motor neurons in the motor cortex and spinal cord, leading to weakness, muscle atrophy, spasticity, and ultimately paralysis and respiratory failure [1,2]. In most cases, ALS is sporadic, but heritability estimates and expanding genetic discovery support a substantial genetic contribution [3,4], with more than 40 genes being involved [5]. Clinically, genetically, and pathologically, ALS overlaps with frontotemporal degeneration (FTD), and a subset of patients exhibit cognitive, behavioral, or executive-function changes, supporting the view that ALS and FTD are part of a broader neurodegenerative disease spectrum [6]. A central molecular hallmark in the overwhelming majority of ALS cases is the pathology of an RNA-binding protein (RBP), TAR DNA-binding protein 43 (TDP-43), including nuclear depletion and cytoplasmic aggregation, which disrupts RNA processing, genome regulation, and chromatin states [7,8,9]. Under physiological conditions, TDP-43 is predominantly nuclear and binds thousands of RNA targets; it regulates multiple steps of RNA metabolism, including pre-mRNA splicing, exon inclusion or exclusion, intron retention, RNA stability, RNA transport, microRNA processing, and stress-granule dynamics [10,11,12]. RNA-processing defects are particularly relevant in neurons because motor neurons rely on long-distance RNA transport, local translation in axons and synapses, and the precise regulation of alternatively spliced transcripts required for synaptic maintenance, cytoskeletal organization, mitochondrial function, and stress responses. In ALS, disrupted RNA processing can lead to cryptic exon inclusion, aberrant splicing, altered polyadenylation, defective RNA localization, and impaired translation of transcripts essential to neuronal survival [13,14]. These changes may contribute to selective motor-neuron vulnerability by reducing the production of functional proteins and increasing the burden of misprocessed or unstable RNAs.

Although mutations in numerous genes have been associated with ALS, pathogenic variants in a small number of these account for most known disease-causing mutations. The most common genetic cause of ALS in the European population is the hexanucleotide repeat expansion (HRE) mutation in the *C9orf72* gene, representing about 40% of familial ALS (fALS) cases and 10% of sporadic ALS (sALS) cases [15,16]. HRE mutations are also associated with FTD. The second most common mutation is in the gene encoding superoxide dismutase 1 (SOD1), which was the first gene to be linked to the disease and one of the best-studied causes of fALS, with up to 24% of fALS cases and 1% of sALS cases [17,18], while mutations in *TARDBP*, the gene encoding TDP-43, and the fused in sarcoma (FUS) gene are responsible for up to 5% of ALS cases [19,20,21]. SOD1 mutations lead to protein misfolding and aggregation, which cause neuronal stress by disrupting protein quality control systems, oxidative stress with mitochondrial damage, ER stress, and axonal transport disruption [18]. ALS-associated FUS mutations frequently impair nuclear import and promote cytoplasmic accumulation, suggesting that disrupted nucleocytoplasmic transport and altered RBP localization represent convergent pathogenic mechanisms across genetically distinct ALS subtypes, with potential consequences for RNA-processing pathways [22,23]. The HRE mutation is thought to cause toxicity and neurodegeneration via three pathogenic mechanisms. The first mechanism involves the haploinsufficiency of C9orf72; the second one is RNA toxicity, in which RNA foci bind and sequester RBPs, consequently affecting post-transcriptional gene regulation [24,25,26]; and the third mechanism refers to repeat-associated non-ATG (RAN) translation, which generates toxic dipeptide repeat polypeptides [27,28,29].

Nuclear TDP-43 loss has been associated with the decondensation of repetitive elements, including LINE retrotransposons, thereby linking transposable element (TE) biology to ALS mechanisms [30,31]. Moreover, three molecular subtypes were identified in cortex samples from ALS patients: one characterized by oxidative and proteotoxic stress, a second by glial activation and inflammation, and a third by retrotransposon reactivation [32]. It was also shown that pathological aggregations of TDP-43 correlate with retrotransposon de-silencing in vivo [32]. Furthermore, TE dysregulation may both reflect and amplify the broader disruption of RNA and genome regulatory networks in ALS and will be discussed in more detail in Section 4.

Transposable elements (TEs) are recognized as major drivers of genome evolution and regulatory innovation and constitute roughly one-half to two-thirds of mammalian genomes, with estimates varying according to the analytical approach [33,34,35]. As mobile DNA elements, they can propagate within genomes without host cell DNA [36,37] and are classified into two groups according to their transposition intermediates. Class I transposable elements are retrotransposons that replicate via RNA intermediates using a “copy and paste” mechanism, while class II DNA transposons move to a new location via a “cut and paste” mechanism [38]. Retrotransposons are further divided into LTR retrotransposons with long terminal repeats (LTRs) and non-LTR elements with a distinct mechanism of retrotransposition. LTR retrotransposons are evolutionarily related to retroviruses, as they share a similar mechanism of replication [39]. Human endogenous retroviruses (HERVs) belong to LTR retrotransposons and represent 8% of the genome [34,40]. Although most of them are considered inactive, reactivation has been identified in several diseased conditions and differentiation [41,42,43].

In humans, the predominant active autonomous retrotransposon is LINE-1, a non-LTR retrotransposon [44]. A large number of LINE-1 copies, 500,000, compose 17% of the human genome, with most of them being truncated at the 5′ site, rearranged, or mutated and therefore being retrotransposition-incompetent [34]. It is estimated that about 1000 copies are full-length; however, only 80–100 remain retrotransposition-competent (RC-L1s). Intriguingly, a subset of those, known as “hot LINE-1s”, is responsible for most of the retrotransposition activity within a human genome [45,46]. A full-length LINE-1 is ~6 kb long and contains a 5′UTR with a promoter, two canonical open reading frames (ORF1 and ORF2), and a 3′UTR with a poly(A) tail. ORF1p is an RNA-binding protein with nucleic acid chaperone activity, whereas ORF2p provides endonuclease (EN) and reverse transcriptase (RT) activities that drive target-primed reverse transcription (TPRT), a mechanism to mobilize retrotransposons around the genome [47,48,49]. Primate LINE-1 elements can additionally express an antisense ORF0 from the 5′UTR that can form fusion transcripts and diversify gene expression [50].

**Figure 1:**
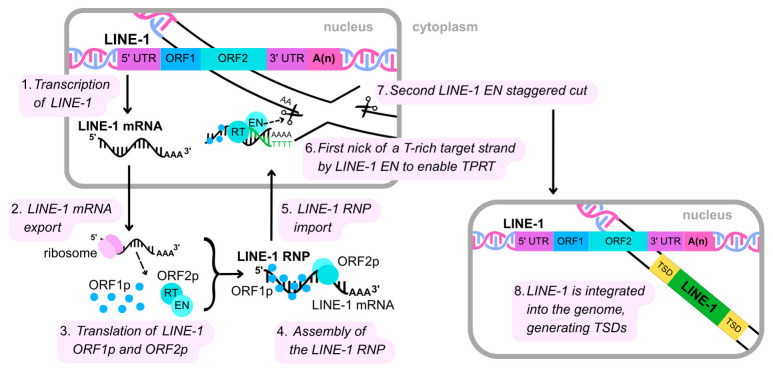
LINE-1 retrotransposition. LINE-1 is transcribed from its 5′UTR promoter (step 1), and the resulting mRNA is translated into ORF1p and ORF2p (step 3). These proteins associate with LINE-1 mRNA to form an RNP (step 4), which gains access to the nucleus (step 5). There, the ORF2p EN introduces a nick at a T-rich target site (step 6), initiating TPRT. The exposed T-rich 3′ DNA end serves as a primer for first-strand cDNA synthesis by the ORF2p RT (green at step 6). EN subsequently introduces a staggered nick in the opposite DNA strand, followed by second-strand cDNA synthesis. Therefore, the integration of the LINE-1 copy results in the formation of target-site duplications (TSDs) flanking the insertion (step 8). TPRT—target-primed reverse transcription; EN—endonuclease; RT—reverse transcriptase; TSD—target-site duplication.

LINE-1 transcription is driven primarily by an internal RNA polymerase II promoter in the 5′UTR, with multiple transcription start sites being influenced by an upstream flanking sequence [51]. The resulting polycistronic mRNA is capped and polyadenylated, exported to the cytoplasm, translated, and assembled with ORF1p/ORF2p into a LINE-1 ribonucleoprotein (RNP) particle [52,53,54,55]. Nuclear entry and retrotransposition show a pronounced cell-cycle bias in dividing cells and involve interactions between the LINE-1 RNP and host nuclear processes [56]. However, LINE-1 retrotransposition has been shown in non-dividing cells, suggesting that active nuclear import is also involved [57]. In the nucleus, ORF2p endonuclease nicks genomic DNA at a consensus T-rich motif, and the exposed 3′ hydroxyl primes reverse transcription from the LINE-1 poly(A) tail, generating cDNA that becomes integrated with characteristic target-site duplications (Figure 1) [47,48,56,58].

Although LINE-1 proteins act preferentially in cis, there is also convincing evidence showing that they help to mobilize nonautonomous retrotransposons such as Alu elements, which are the most abundant human short interspersed nuclear elements (SINEs), SINE-VNTR-Alu elements (SVAs), and mature cellular mRNAs to form processed pseudogenes [59,60,61,62,63]. Despite a small number of active LINE-1s, individuals differ markedly in which polymorphic RC-L1 loci they carry and in the RC-L1 load in their genomes. Because RC-L1 loci differ in both activities of “hot LINE-1s” and genomic context, RC-L1 burden represents a plausible genetic factor that influences disease risk or phenotype, particularly in disorders characterized by sensitivity to genomic regulation and inflammation [45,64].

Even though the broader association of TE with neurodegeneration has been discussed in several reviews, here, we provide an updated and more focused perspective on LINE-1 in ALS. The 2019 review by Savage et al. primarily discussed retrotransposons as a broad class of genomic elements and summarized the initial evidence linking transposable element dysregulation to ALS pathology [65]. A 2021 review expanded this concept to neurodegenerative diseases in general, emphasizing age-related loss of transposable element repression, genomic instability, and neuroinflammation [66]. More recently, a 2024 review further consolidated evidence implicating transposable elements in ALS and related disorders, with the majority of evidence in the ALS section relating to HERVs [67]. In contrast, our review specifically focuses on LINE-1 and integrates recent advances in TDP-43-associated chromatin decondensation and retrotransposon activation in ALS subtypes. It also highlights germline RC-L1 burden in ALS and the interplay of genetic, epigenetic, and immune mechanisms as key differentiating features.

## 2. Host Restriction of LINE-1: Layers of Repression and Surveillance

De novo insertions of LINE-1 or Alu elements are estimated to occur at frequencies of approximately one per 20–200 and 20–40 live births, respectively [68,69,70]. In addition to insertions of LINE-1, Alus, SVAs, and processed pseudogenes, LINE-1 activity is also responsible for substantial genome rearrangements, including deletions, insertions, duplications, and translocations, often mediated by the recombination of conserved retrotransposon sequences [68,71,72,73]. Because uncontrolled retrotransposition threatens genome integrity, mammalian cells have evolved multiple layers of restriction mechanisms. These include DNA methylation and repressive chromatin, RNA processing and truncation, RNA interference-linked pathways, and restriction by RNA- and protein-binding factors [74,75,76,77,78,79]. There is evidence on the host regulation of the LINE-1 retrotransposition cycle at transcription, post-transcription, post-translation, and integration.

Methylation at CpG-rich regions within LINE-1 promoters contributes to transcriptional repression and can vary with age and disease [74,80,81]. Another mechanism of epigenetic inhibition of LINE-1 transcription is histone modification, particularly H3K9me3 and histone H3 deacetylation [82,83,84,85,86,87,88]. Chromatin reader MeCP2 and promyelocytic leukemia zinc finger protein (PLZF) also participate in LINE-1 silencing [89,90]. Some transcription factors, including SOX2 and the tumor suppressor p53, prevent LINE-1 transcription [57,91].

Post-transcriptionally, premature polyadenylation and splicing generate truncated LINE-1 transcripts that are generally incompetent for retrotransposition [76,77]. RNA silencing pathways have evolved as host defenses against mobile genetic elements [92]. piRNAs are active in germ cells, whereas siRNAs and miRNAs function mainly in somatic cells [93,94,95]. For example, the Microprocessor complex involved in miRNA biogenesis can process LINE-1-derived RNAs and reduce LINE-1 activity, supporting a direct connection between small-RNA biogenesis and retrotransposon control [78]. More broadly, the post-transcriptional regulation of retrotransposons includes nuclear RNA surveillance and RNA decay pathways that prevent the accumulation of aberrant or excess retroelement transcripts [79]. In addition, deaminase ADAR and AID/APOBEC families inhibit retrotransposon activity [96,97,98,99,100]. Other protein interactors may reduce LINE-1 RNA and/or LINE-1 proteins [101,102,103].

The post-translational regulation of LINE-1 is linked to interferon-stimulated genes, which mediate the type I interferon response in somatic cells [104,105]. Several inhibitory mechanisms target ORF1p [106] or ORF2p [107], where some pathways lead to the formation of stress granules (SGs) that include LINE-1 proteins and RNA [108,109]. Components of SGs may subsequently be removed through autophagy [110,111].

Furthermore, de novo retrotransposon insertions can trigger heterochromatization by binding to heterochromatin formation factors or components of the Human Silencing Hub complex [55,85,112,113,114]. Another important repression mechanism involves repair factors such as BRCA1 and ERCC1/XPF from host DNA repair machinery [115,116,117].

Overall, host factors regulate LINE-1 propagation at every level of the retrotransposition cycle [72,118]. In the nucleus, epigenetic regulators and transcription repressors limit LINE-1 expression, while integration events are prevented by host DNA repair factors related or not to DNA breaks and by heterochromatization. Cytoplasmic host defense mechanisms include RNA silencing, type I IFN response, and the sequestration of LINE-1 mRNA, ORF1p, and ORF2p into membraneless organelles, potentially followed by autophagic degradation. Nevertheless, LINE-1 and nonautonomous elements can evade host cellular regulatory mechanisms, causing genetic diseases [119,120]. Elevated LINE-1 expression is a hallmark of many cancers [73,121,122]; moreover, LINE-1 retrotransposition has also been shown to contribute to neuronal mosaicism and the development of neurological disorders [123,124].

## 3. LINE-1 Activity in the Nervous System

Retrotransposons are increasingly recognized as important contributors to neurodevelopment, aging, and neurological diseases. More than 20 years ago, considerable attention was drawn to the discovery of LINE-1 retrotransposition in neural progenitor cells (NPCs) [57]. Since then, numerous studies have demonstrated LINE-1 activity in human embryonic stem cells (hESCs), neural stem cells, NPCs, and mature neurons [80,125,126], suggesting that LINE-1 plays an important role in neurogenesis and neuronal diversification. Somatic LINE-1 insertions preferentially target genes associated with neurogenesis and synaptic function compared with germline insertions [127], further supporting the importance of LINE-1 activity in the nervous system. Notably, the human brain is the only healthy somatic tissue where LINE-1s are de-repressed [80,128].

Several studies have estimated that neurons harbor approximately 1 to 13 somatic insertions of LINE-1 per cell, although estimates vary depending on the experimental approach and the cell type analyzed [125,129,130]. Hippocampal neurons generally exhibit the highest levels of somatic retrotransposition [80,125]. These findings support the concept that genetically, the human brain is a mosaic at multiple scales. Somatic mutations can generate neuronal genomic diversity, and the developmental timing of mutational events determines whether mosaicism is germline, somatic, or mixed.

Somatic genome mosaicism occurs in both normal and pathological tissues during development and aging, encompassing a variety of mutations ranging from single-nucleotide variants to chromosomal alterations [131]. In neural stem and progenitor cells, transcriptional programs that maintain multipotency are associated with the repression of LINE-1 through RNA interference and chromatin control. In contrast, the de-repression of LINE-1 transcription is related to the differentiation of NPCs [132,133,134]. Indeed, LINE-1 activation has been shown to upregulate numerous genes that are involved in neuronal differentiation [135]. Transposable elements, including LINE and SINE families, have been extensively co-opted into regulatory processes underlying neuronal differentiation and mammalian brain evolution [136]. There is particularly strong evidence for the exaptation of SINEs in primates. For example, primate-specific ncRNA originated from Alus [137,138], while Alu-derived sequences located in the 3′UTR of the primate-specific transcript isoform can function as miRNA sponges [139,140].

Valuable insights into the role of LINE-1 elements in brain function and evolution emerged from single-neuron studies [125,127,129,130,141], which demonstrated somatic insertions in the developing cortex. In addition to retrotransposition itself, LINE-1-derived transcripts contribute to the complexity of the human-specific transcriptome. One such example is *LINC01876*, an lncRNA of LINE-1 origin [142]. Moreover, regulated LINE-1 expression may help protect NPCs from premature differentiation, which could play a role in the neotenic characteristics of the human brain [123,143,144].

Overall, LINE-1 elements influence neuronal biology at two distinct levels: through insertions and insertion-independent effects mediated by LINE-1 RNA transcripts and LINE-1-encoded proteins. RC-L1 insertions contribute to genetic instability by creating deletions at target sites, mobilizing flanking DNA, recombining with other retrotransposons, and inducing chromosomal inversions and interchromosomal translocations [124]. LINE-1 activity has also been associated with non-allelic homologous recombination and the formation of DNA double-strand breaks (DSBs). ORF2p endonuclease activity can directly induce DSBs, whereas indirect mechanisms involving replication stress and DNA repair may further exacerbate genomic damage [145,146]. Even retrotransposition-incompetent LINE-1 loci may contribute to genomic instability through aberrant ORF2p fragments or recombination between repeats [147].

Somatic LINE-1-associated structural variation has been detected in the nervous system, and LINE-1-rich regions appear to act as hotspots for somatic deletions and copy number variation [130]. Such DNA damage and structural variations resemble aging-associated molecular signatures commonly observed in neurodegenerative disorders, including the accumulation of genomic lesions and impaired DNA repair capacity [148]. Furthermore, the overexpression or mislocalization of LINE-1 ORF1p can induce cytotoxicity and disrupt normal RNA or protein degradation pathways, promoting apoptosis, cellular senescence, or inflammatory immune responses—hallmarks of many neurological diseases [149]. LINE-1 de-repression and increased levels of LINE-1 RNA transcripts are also characteristic of many neurological disorders caused by mutations in regulatory genes. A notable example is Rett syndrome, in which a pathogenic mutation in MeCP2 impairs binding to methylated CpG islands within LINE-1, resulting in abnormal LINE-1 activation [89,107,150,151].

## 4. LINE1 Retrotransposons in ALS

Different retrotransposons, including non-LTR LINE and SINE elements, and LTR HERVs, have been implicated in the development of ALS, predominantly in association with TDP-43 pathology [65]. However, elevated levels of retrotransposons’ transcripts were also linked to C9orf72-associated ALS/FTD pathology [152,153].

Regarding non-LTR retrotransposons, it has been shown that TDP-43 directly binds transcripts of LINE-1 and Alu origin [106], whereas SVAs may facilitate ALS indirectly through exonization events [154]. The loss of nuclear TDP-43 drives retrotransposon activation, which has been shown to contribute to neurodegeneration in two Drosophila models and a mouse model of ALS [155,156,157]. Similar observations have been reported in human ALS nuclei without TDP-43, as the decondensation of chromatin occurred together with increased LINE-1 activity [30]. Arguably, a reduced methylation pattern of RC-L1 elements and chromatin decondensation lead to the de-repression of LINE-1 elements [30,74]. In addition to elevated levels of LINE-1 transcripts, polymorphic insertions have also been detected in ALS patients [158,159]. Retrotransposon insertions might change the regulation of endogenous gene expression, causing loss-of-function somatic mutations. Moreover, ALS-associated proteins colocalized with LINE-1 ORF1p in cytoplasmic membraneless granules, while the overexpression of some of those proteins inhibited LINE-1 retrotransposition in cell cultures [149]. The same study also showed minimal altered LINE-1 expression in sporadic ALS tissues. Collectively, these findings suggest that LINE-1 and nonautonomous elements may be involved in neuronal dysfunction and neurodegeneration related to ALS.

Although this review focuses on LINE-1 elements in association with ALS, it is also important to mention HERVs, another group of retrotransposons that differ from LINE-1s in both structure and mechanism of mobilization. HERVs have been strongly implicated in ALS, as they are transcriptionally active in pathological conditions, and their transcripts have been shown to colocalize with TDP-43 RNA in neurons from ALS patients. Furthermore, the expression of the HERV-K envelope protein is elevated in post-mortem brain tissue [160,161]. In serum and cerebrospinal fluid from ALS patients, antibody levels against fragments of HERV-K env significantly correlate with clinical measures of disease severity, suggesting their potential as early biomarkers for ALS [162]. Notably, HERVs provide some of the strongest human evidence linking transposable element dysregulation to ALS.

**Figure 2:**
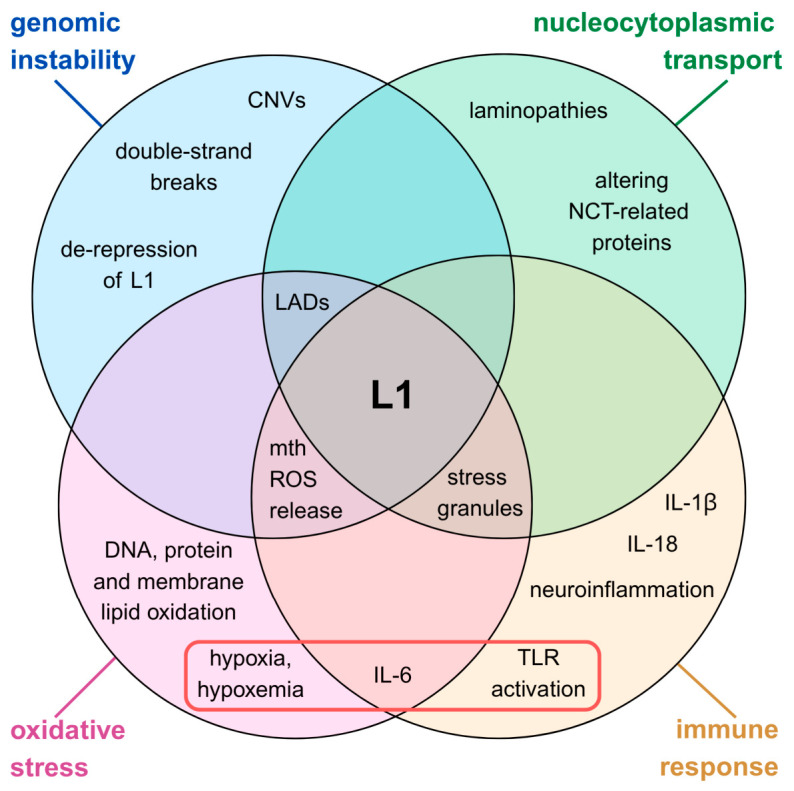
Pathological molecular mechanisms of ALS linked to LINE-1 activity. The Venn diagram depicts the pathological ALS mechanisms and how their key players overlap and involve LINE-1. Hypoxia, hypoxemia, IL-6, and TLR activation are consequences of neurodegeneration that generate a positive feedback loop in disease, leading to more damage (circled in red). Further explanations are in the main text and referenced in Table 1. DSB—double-strand break; CNV—copy number variant; LAD—lamina-associated domain; NCT—nucleocytoplasmic transport; ROS—reactive oxygen species; TLR—Toll-like receptor.

ALS pathogenesis can be conceptualized as the convergence of genome instability, oxidative stress, nucleocytoplasmic transport defects, and neuroinflammation. LINE-1 biology intersects with each of these axes through (i) retrotransposition-dependent DNA damage and genome remodeling, (ii) the retrotransposition-independent toxicity of LINE-1 RNA and LINE-1 proteins, and (iii) innate immune activation by LINE-1-derived nucleic acids (Figure 2).

### 4.1. RC-L1 Burden as a Genetic Risk Factor in Sporadic ALS

One important recent advance is the demonstration that ALS genomes carry an increased burden of RC-L1 alleles at polymorphic loci, with at least 46 RC-L1 loci showing the strongest association with disease risk [161]. Notably, this association is not driven by a single causative insertion but instead reflects the cumulative contribution of multiple active LINE-1 loci, providing strong evidence for a polygenic component of ALS susceptibility. Interestingly, only a single LINE-1 locus was associated with earlier disease onset in a study analyzing whole-genome sequences from the New York Genome Center ALS consortium. Together, these genetic findings extend previous studies that cataloged retrotransposon insertion polymorphisms in ALS cohorts [161,162] and suggest that RC-L1 burden may contribute to missing heritability in sporadic ALS.

The mechanisms through which increased RC-L1 burden influences ALS risk remain to be established experimentally. Based on current knowledge of LINE-1 biology, a greater number of active LINE-1 loci could increase the likelihood of (i) somatic retrotransposition events during development or aging, (ii) elevated basal levels of LINE-1 RNA and proteins under conditions of partial de-repression, and (iii) enhanced chronic innate immune activation triggered by LINE-1-derived nucleic acids, particularly during age-related decline in epigenetic repression. Although each of these mechanisms is biologically plausible, their direct contribution to ALS pathogenesis has not yet been demonstrated.

### 4.2. Retrotransposition-Independent Toxicity in ALS: LINE-1 RNA and LINE-1 Proteins

The loss of nuclear TDP-43 is associated with the decondensation of LINE elements shown in post-mortem human FTD-ALS brain [30] and more general TDP-43-linked chromatin dysfunction [9,31]. The disruption of TDP-43-dependent nuclear RNA surveillance and retrotransposon repression results in the accumulation of LINE-1 transcripts and chromatin decondensation at LINE-1 loci in ALS mouse models, leading to widespread transcriptional dysregulation that occurs independently of new retrotransposition events [30,156,163]. Further evidence of LINE-1 RNA function in the nucleus involves antisense LINE-1 transcripts, which interact with the nuclear matrix protein Matrin-3 to directly influence chromatin organization in mouse hepatocytes. Furthermore, a network formed by antisense LINE-1 RNA and ALS-associated mutant Matrin-3 leads to an abnormal distribution of H3K27me3, a key epigenetic marker associated with heterochromatin formation. This disruption may consequently alter the three-dimensional organization of chromatin [164,165]. In TDP-43 pathology, failure of nuclear RNA surveillance could allow LINE-1 RNAs to accumulate in the nucleus, where they can alter chromatin architecture and transcriptional control, generating global dysregulation without requiring productive retrotransposition. This reframes LINE-1 from a purely “mutagenic insertion” agent to a regulatory and architectural stressor in diseased neurons.

Beyond RNA, LINE-1 proteins can perturb neuronal homeostasis. A recent study demonstrated that oxidative stress could drive ORF1p nuclear translocation in human neuronal models via direct interactions with nuclear import machinery (KPNB1), nuclear pore components (NUP153), and nuclear lamina proteins (Lamin B1). Nuclear ORF1p disrupted nuclear envelope integrity, nucleocytoplasmic transport, and heterochromatin structure—features strongly implicated in neurodegeneration and aging [166]. These data complement extensive evidence showing that nucleocytoplasmic transport defects are central to ALS/FTD pathobiology and can be exacerbated by TDP-43 pathology [10,167,168,169,170]. Together, these findings support a model in which LINE-1 de-repression and proteostasis stress can amplify nuclear dysfunction through both RNA- and protein-mediated pathways.

### 4.3. Innate Immunity and Neuroinflammation: The cGAS–STING Axis as a Mechanistic Bridge

Neuroinflammation is increasingly recognized as both a driver and amplifier of ALS progression [171]. Innate immune sensors detect nucleic acids through endosomal and cytosolic receptors, but they only partially discriminate between pathogen-derived and endogenous nucleic acids [105]. When LINE-1 is de-repressed, ORF2p-mediated reverse transcription can generate aberrant DNA species and DNA/RNA hybrids that may be mislocalized in the cytosol, functioning as damage-associated molecular patterns (DAMPs) [145,146,172,173]. It was also shown that DNA damage or impaired DNA repair mechanisms stimulate the export of LINE-1 DNA into the cytoplasm [105,174].

Cytosolic DNA or RNA-DNA hybrids initiate the cellular defense mechanism of the cyclic GMP-AMP synthase–stimulator of interferon genes (cGAS–STING) pathway. Downstream from STING, TANK-binding kinase 1 (TBK1) is recruited to phosphorylate interferon regulatory factor 3 (IRF3), which is translocated to the nucleus to induce the expression of various interferons (IFNs) and interferon-stimulated genes [48,175]. In addition, STING activates the NF-κB pathway, leading to the production of inflammatory cytokines and chemokines such as tumor necrosis factor (TNF), interleukin-1β (IL-1β), and interleukin-6 (IL-6) [176]. Three prime repair exonuclease 1 (TREX1) targets ssDNA and dsDNA to limit the cGAS-STING-mediated interferon response [150,176].

The cGAS–STING pathway detects cytosolic DNA originating from multiple sources, including endogenous retrotransposons and released genomic and mitochondrial DNA [177]. Activation of this pathway has been implicated in ALS mouse models and in cortical and spinal motor neurons from patients with ALS [178,179,180,181,182], which induces the type I IFN response in ALS cases [183]. However, a recent integrated transcriptomic study in an ALS SOD1 model and in human ALS tissue identified STING–TBK1 signaling as a driver of a type II IFN pathway [184]. Additional evidence comes from studies of downstream pathway components. In particular, mutations in TANK-binding kinase 1 (TBK1) have been implicated in ALS [185,186,187]; these mutations result in impaired mitophagy and autophagy, as well as the accumulation of protein aggregates [188,189]. The release of mitochondrial DNA and other danger-associated molecules activates the cGAS–STING pathway, which induces chronic inflammatory signaling [175], thereby promoting protein aggregation, amplifying neuroinflammation, and aggravating neurodegeneration, including ALS [173,190].

The induction of cGAS-STING signaling with LINE-1-derived nucleic acids stimulates downstream TBK1/IRF and NF-κB signaling pathways, producing interferon programs, microglial activation, and inflammatory reinforcement in ALS. Inflammatory markers drive differentiated helper T cells to the central nervous system, where they recognize ORF1p, secrete neurotoxic granzyme B, and also kill neighboring neurons, which further release ORF1p, thereby amplifying neuronal loss [191]. This model is consistent with genetic and functional links between ALS and innate immunity regulators (e.g., TBK1) and with the emerging view that sterile infection states can arise from endogenous nucleic acid dysregulation [105,175,184]. However, one study also showed that cGAS localizes to the nucleus and may upregulate LINE-1 transcription by binding to promoters of the LINE-1 transcription factors RUNX3 and CTCF, indicating that non-canonical pathways exist in human cells [192]. An important suppressor of LINE-1 is also TREX1. The silencing of this exonuclease leads to an increased accumulation of LINE-1-derived nucleic acids, which subsequently activate cGAS–STING signaling [105,173,193]. Thus, LINE-1 appears to be an important contributor to cGAS–STING pathway activation; however, the extent to which this mechanism contributes to ALS pathology remains unclear.

### 4.4. Oxidative Stress and Feed-Forward Amplification

Oxidative stress is a prominent feature of ALS and other neurodegenerative disorders driven by mitochondrial dysfunction, chronic inflammation, and the high metabolic demand of motor neurons [194,195,196,197]. Induced oxidative stress has also been shown to promote the formation of larger ORF1p stress granules [149], which could indicate potential retrotransposon defense. In this process, the cell supposedly seizes active and available ORF1p proteins and their bound LINE-1 RNA molecules to reduce their nuclear import and retrotransposition. Oxidative stress can simultaneously (i) weaken epigenetic repression, (ii) promote LINE-1 protein relocalization and stress-granule biology, and (iii) increase the generation of damaged nucleic acids, which amplify innate immune activation. Extrapolating from this, oxidative stress provides a plausible feed-forward loop in which LINE-1 de-repression and inflammatory signaling mutually reinforce oxidative stress, progressively eroding nuclear and genomic homeostasis.

ALS pathogenesis related to LINE-1 activity is summarized in Table 1.

**Table 1:** LINE-1-linked molecular species and inferred ALS-relevant consequences. Examples include LINE-1 RNA (chromatin accessibility increase and transcriptional amplification), ORF1p (nuclear envelope and nucleocytoplasmic transport disruption), ORF2p/RT-derived intermediates (DNA damage and cytosolic DNA accumulation), and downstream innate immune activation (interferon programs and microglial activation). HR—homologous recombination; DSB—double-strand break; CNV—copy number variant; LAD—lamina-associated domain; TLR—Toll-like receptor; ROS—reactive oxygen species; NCT—nucleocytoplasmic transport.

PROCESS	CAUSATIVE LINE-1 PARTICLE Or ACTIVITY	OUTCOME	SOURCE
genomic instability	LINE-1 insertion	deletions and insertions	[124]
		chromosomal inversions	
		interchromosomal translocations	
		non-allelic HR	[147]
		DSBs	
	non-RC-L1	mutated and truncated products are cytotoxic and can cause DSBs	[147]
	ORF0	enhances LINE-1 activity	[124]
		possible toxic protein fusions	[50]
	ORF1p	alters normal RNA and protein degradation	[149]
	ORF2p	CNVs	[130]
		DSBs	[149]
immune response	LINE-1 mRNA, ORF1p, ORF2p, LINE-1 RNP	sterile infection	[77]
		neuroinflammation	[196]
		microglia activation (M1), release of IL-1β and IL-18	[198,199,200]
	LINE-1 mRNA	TLR activation	[79]
		IL-6 production	[201]
	ORF1p	release of granzyme B	[191]
oxidative stress	via neuroinflammation	mitochondria release ROS and initiate transcription of LINE-1	[202]
		ORF1p stress granule formation	[149]
nucleocytoplasmic transport	ORF1p	laminopathy	[166]
		de-repression of LINE-1 transcription via LADs	
		alteration in NCT proteins	[166,169]

## 5. Therapeutic Implications

LINE-1 biology is particularly relevant to the multilevel nature of ALS, as LINE-1 dysregulation has the potential to influence multiple pathogenic processes simultaneously, including chromatin organization, RNA metabolism, innate immune signaling, genomic stability, and neuronal function. Consequently, LINE-1 and its associated pathways may represent promising biomarkers and therapeutic targets in ALS. A major advantage of biomarker-based approaches is the possibility of non-invasive or minimally invasive sampling. LINE-1-derived nucleic acids can be detected in blood and cerebrospinal fluid and have already shown diagnostic and prognostic value in several diseases, particularly cancer [192,193,194,195,196]. Given the involvement of LINE-1 elements in multiple processes linked to ALS pathogenesis, LINE-1-based biomarkers could aid patient stratification, disease monitoring, and prognosis. Ultimately, such markers may improve our understanding of ALS heterogeneity and support the development of personalized therapeutic approaches.

The growing evidence suggesting a role of LINE-1-derived nucleic acids in the activation of innate immune responses could lead to therapeutic approaches that should reduce the accumulation of cytosolic LINE-1 DNA and DNA–RNA hybrids [48]. The clearance of these nucleic acid species may prevent the activation of nucleic acid-sensing pathways and consequently attenuate chronic neuroinflammation.

Another promising therapeutic strategy involves enhancing the activity of endogenous proteins that normally suppress LINE-1 expression. Such an approach may be particularly beneficial in cases where the function or expression of these suppressors is impaired, as restoring their activity could help re-establish the physiological control of LINE-1 elements. However, the indiscriminate enhancement of LINE-1 suppressors may not always be advantageous. Many of these proteins participate in multiple cellular processes beyond transposable element regulation, and excessive activation could potentially disrupt normal cellular functions or produce unintended off-target effects. Therefore, the precise and controlled modulation of those suppressors is essential. Future studies should aim to determine the optimal degree of enhancement required to restore LINE-1 homeostasis while minimizing potential adverse consequences.

Overall, the findings suggest the following future research directions:

1. RC-L1 allele burden may be associated with molecular markers such as elevated LINE-1 RNA expression, interferon signaling, and indicators of DNA damage. Therefore, individuals carrying a higher number of active RC-L1 elements may represent a genetically predisposed subset of sporadic ALS patients [158,159]. Identifying such patients could improve diagnosis and prognosis and help guide the development of targeted therapeutic approaches.
2. The pharmacologic or genetic modulation of the cGAS–STING axis or downstream TBK1-dependent inflammatory programs may reduce LINE-1-linked neuroinflammation, with cell-type specificity likely critical to efficacy and safety [175,184], indicating the innate immune pathway as a therapeutic target.
3. RNA burden causes failure of nuclear RNA surveillance, chromatin maintenance, nucleocytoplasmic transport, and proteostasis [9,30,166]; therefore, RNA surveillance or more specific RNA degradation could be a potential therapeutic strategy in TDP-43-related diseases.
4. Conversely, therapeutic benefit may be achievable by reducing LINE-1 RNA/protein toxicity through the use of reverse transcriptase inhibitors or epigenetic modulators that restore LINE-1 silencing. The detailed structure of ORF2p has recently been determined, enhancing the possibility of designing specific targets [48,58].

Addressing these questions will advance our understanding of the complex relationship between LINE-1 dysregulation and ALS. Together with continued progress in the development of improved experimental techniques and bioinformatic tools, this will open new avenues for identifying potential therapeutic strategies. However, it is important to recognize the current limitations, including off-target effects and the limited success of translating the approaches discussed above into clinical applications. Consequently, transposable element (TE)-targeted therapies remain at an early stage of development.

## 6. Conclusions and Challenges

ALS is shaped by interacting genetic and age-associated processes that also perturb nuclear and genomic homeostasis and ignite neuroinflammation. LINE-1 retrotransposons connect these layers through three mechanistic routes: (i) genetic variability in RC-L1 burden as a quantitative risk factor in sporadic ALS; (ii) the retrotransposition-independent toxicity of LINE-1 RNA, which promotes chromatin opening and epigenetic noise when nuclear RNA surveillance fails in TDP-43 pathology; and (iii) innate immune activation, plausibly via cGAS–STING, driven by LINE-1-derived nucleic acids and reinforced by oxidative stress. Integrating these concepts supports a unifying model in which germline RC-L1 load sets vulnerability, while disease-linked failures of chromatin and RNA control unleash LINE-1-derived molecular triggers that amplify nuclear dysfunction and neuroinflammation.

There are still substantial gaps in our understanding of how LINE-1 activity is mechanistically linked to ALS pathogenesis. A primary unresolved question is whether LINE-1 dysregulation acts as a causal driver of disease or represents a downstream consequence of neurodegeneration. Resolving this “driver versus passenger” relationship will require more detailed mechanistic insights into LINE-1 regulation across disease stages and different cell types. Future progress is likely to come from the application of single-cell multi-omics approaches and long-read sequencing technologies, which enable the resolution of cell-type-specific transcriptional changes, full-length LINE-1 transcripts, and structural variation in nucleic acids. Although these technologies are already available, major challenges remaining are the integrative analysis of large-scale datasets and additional issues of selecting appropriate sample sizes, disease stages, and anatomically relevant CNS regions.

Another important open question concerns the identification of functionally relevant LINE-1 loci. Not all LINE-1 elements are equally active nor are equally disease relevant. In this context, further investigation of higher-order chromatin architecture may be particularly informative. For example, the analysis of topologically associating domains (TADs) and their potential disruption in ALS could provide insights into how LINE-1 elements are embedded within or influence three-dimensional genome organization.

Therapeutically, targeted strategies aimed at suppressing transcriptionally active LINE-1 loci represent an emerging point of interest. In principle, locus-specific genome editing approaches, including CRISPR-based systems, could be explored to selectively silence active retrotransposons, although significant technical and safety challenges remain.

A deeper understanding is also needed regarding the interplay between LINE-1 elements and key ALS-associated proteins, including TDP-43 and FUS, in the context of nucleocytoplasmic transport. Furthermore, the role of membraneless organelles, such as stress granules, remains incompletely understood; it is unclear whether their formation represents a protective stress response or contributes directly to pathology through the sequestration of RNA and RNA-binding proteins. This is not only relevant in the cytoplasm but also in the nucleus, where ALS-associated expanded repeat (HRE) transcripts participate in phase-separated condensates and thereby influence cellular homeostasis [203,204].

Finally, given the substantial interindividual variability in LINE-1 insertions and transcriptional activity, future studies should place greater emphasis on individual analyses. Such approaches may improve our understanding of patient-specific disease mechanisms and facilitate the development of precision medicine approaches.

Collectively, the available evidence supports an important role for LINE-1 in maintaining cellular homeostasis and implicates its dysregulation in ALS pathobiology. Nevertheless, ALS is a heterogeneous, multifactorial neurodegenerative disorder driven by convergent and interacting molecular, cellular, and genetic mechanisms; therefore, LINE-1 should be viewed as one factor of a broader pathogenic network that may influence disease initiation, progression, or subtype-specific vulnerability. Although LINE-1 dysregulation is likely one component of a multifactorial pathogenic network, a more detailed understanding of LINE-1 function across physiological and disease-relevant contexts may therefore refine mechanistic models of ALS, enable biomarker discovery, and reveal therapeutic opportunities for molecularly defined ALS subtypes.

## Data Availability

No new data were created or analyzed in this study. Data sharing is not applicable to this article.

## Abbreviations

The following abbreviations are used in this manuscript:

ALS	Amyotrophic lateral sclerosis
cGAS–STING	Cyclic GMP-AMP synthase–stimulator of interferon genes
CNV	Copy number variant
DAMP	Damage-associated molecular pattern
DSB	Double-strand break
FTD	Frontotemporal degeneration
FUS	Fused in sarcoma
HERV	Human endogenous retrovirus
hESC	Human embryonic stem cells
HRE	Hexanucleotide repeat expansion
LAD	Lamina-associated domain
IFN	Interferon
IL	Interleukin
IRF	Interferon regulatory factors
LINE-1	Long interspersed nuclear element-1
LTR	Long terminal repeats
NCT	Nucleocytoplasmic transport
NPC	Neural progenitor cells
ORF	Open reading frame
RBP	RNA-binding protein
RC-L1	Retrotransposition-competent LINE-1
ROS	Reactive oxygen species
RT	Reverse transcriptase
SINE	Short interspersed nuclear element
TBK1	TANK-binding kinase 1
TDP-43	TAR DNA-binding protein 43
TE	Transposable element
TLR	Toll-like receptor
TPRT	Target-primed reverse transcription
TREX1	Three prime repair exonuclease 1
